# A Set of Yellow Mustard (*Sinapis alba* L.) Germplasm with Polycotyledony

**DOI:** 10.3390/plants12233919

**Published:** 2023-11-21

**Authors:** Yong-Bi Fu

**Affiliations:** Plant Gene Resources of Canada, Saskatoon Research and Development Centre, Agriculture and Agri-Food Canada, 107 Science Place, Saskatoon, SK S7N 0X2, Canada; yong-bi.fu@agr.gc.ca

**Keywords:** polycotyledonous germplasm, inheritance of polycotyledony, tricot, tetracot, tricot breeding, yellow mustard

## Abstract

A world collection of 132 yellow mustard (*Sinapis alba* L.) accessions was characterized in a greenhouse to identify germplasm with polycotyledony and to assess the genetic segregation of tricot and tetracot seedlings in selfed S1 and S2 generations. The effort identified a set of 46 yellow mustard accessions with frequent occurrences of polycotyledony. The revealed genetic segregations seemed to suggest the development of tricot and tetracot seedlings in yellow mustard was largely controlled by a combination of genes at multiple diallelic loci. The identified tricot germplasm can be used to facilitate the genetic and/or genomic analysis of polycotyledonous germplasm for a better understanding of genetic and developmental mechanisms conditioning polycotyledony and the development of yellow mustard lines for explorable tricot breeding.

## 1. Introduction

Polycotyledons, which are plants that have more than two cotyledons, have been long known as a sporadic occurrence in dicotyledonous plants [1] and are commonly treated as a rare and trivial abnormality [2]. However, the exploration of polycotyledon use in plant breeding is not lacking in the literature [3,4,5,6]. For example, Griffith [7] reported that the tetracotylous and tricotylous seedlings of *Cupressus lusitanica* Mill. had significantly greater one-year heights than dicotyledonous seedlings. Venkatesh and Sharma [8] reported that the tricotylous *Eucalyptus* L’Hér. seedlings are more vigorous than dicotylous seedlings. Raiora and Zsuffa [9] explored the potential of using atypical *Populus* L. seedlings in poplar breeding. Rick et al. [5] developed a polycot-based seedling marker for tomato breeding and Madishetty et al. [10] identified another polycot allele that was involved with the negative regulation of polar auxin transport conditioning the development of the polycotyledony. It was found that the *Arabidopsis* cotyledons influenced the amount and proportion of hybrid vigor in total plant growth [11] and that tricotyledony in sand rice (*Agriophyllum squarrosum*) was associated with increased seed yield [12]. Taylor and Mundel [13] released a multiple-cotyledon red clover genetic marker stock: L38-1485. Hu et al. [6,14] identified a tricot mutant from a BC3F2 sulphonylurea herbicide-resistant population derived from three *Helianthus annuus* L. inbred lines and a wild *H. annuus* strain and developed sunflower germplasm called ‘Tricot’ with up to 50% tricot penetrance (USDA-GRIN PI642084).

Early genetic analyses indicate that polycotyledony is a heritable trait [2,15] and follows polygenic inheritances [16,17,18,19]. The last 20 years have seen increased research on polycotyledonous mutants in *Arabidopsis* Heynh. due to the release of the *Arabidopsis thaliana* (L.) Heynh. genome. This research helped to identify and characterize many loci conditioning the development of polycotyledony in *Arabidopsis*, allowing for a better understanding of polycotyledony origin and development [20]. However, such in-depth research is lacking for other dicotyledonous plant species [20].

The polycotyledony in yellow mustard has long been documented (e.g., see [21]), but little research has been done on yellow mustard polycotyledons. The Plant Gene Resources of Canada (PGRC; the Canadian national seed GenBank at Saskatoon) maintains a small world collection of 134 yellow mustard (*Sinapis alba* L.) accessions. These accessions were collected over several decades from more than 23 countries representing mainly Europe, Asia, and North America (Table S1). The collection consists of cultivars, breeding lines, landraces, and wild accessions with yellow or brown seed colors and has been characterized by its genetic diversity [22,23]. Here, we report findings from an effort to characterize the yellow mustard germplasm with polycotyledony. Specifically, 132 accessions were grown in a greenhouse and the surviving polycotyledonous seedlings were characterized. The identified polycotyledonous plants were selfed with bags and selfed seeds were grown to evaluate polycotyledon segregation. Extra effort was also made to generate the S2 progeny for further genetic segregation analysis.

## 2. Results and Discussion

Three sets of greenhouse characterization revealed 89 tricot seedlings from 46 (out of 132; 34.8%) accessions (Table 1 and Table S1). The frequencies of tricot occurrence in these identified accessions ranged from 0.59 to 11.25% with an average of 1.93% of the germinated seeds. However, when the total seeds planted were considered, the occurrence frequencies were slightly lower, ranging from 0.53 to 7.14% and averaging 1.53%. Note that the number of seeds planted or germinated seeds per accession ranged from 90 to 198 or from 11 to 188, respectively (see Table S1). Tricot occurrence varied among the three sets of assessment; only two accessions (CN102136 and CN102152) had tricots in all three sets, while most of them showed up in one or two sets. Note that the germination rates for these accessions varied greatly within each set, but overall were high for the three sets of assessments, ranging from 77.2% to 85.4% even with variable seeds planted for each set (Table 1). Also, the tricot occurrence did not seem to be associated with seed color (yellow or brown), cultivar or landrace, or origin of country or region (Table 1). No tetracots were detected in these three sets of assessments.

**Figure 1:**
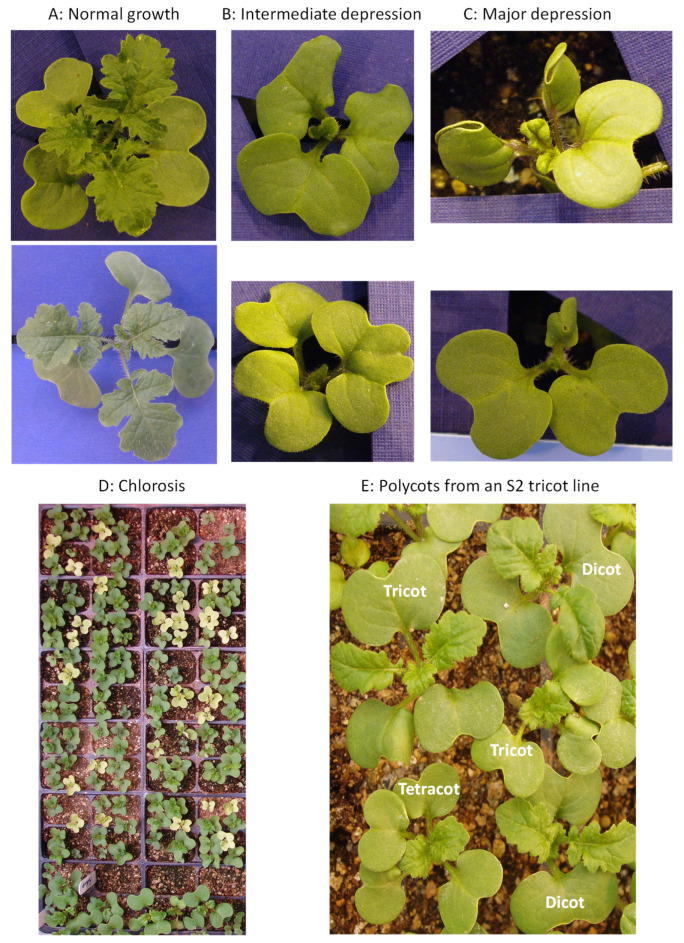
Illustration of polycot seedling growth patterns: (**A**) normal growth; (**B**) intermediate depression; (**C**) major depression; (**D**) seedlings with chlorosis; and (**E**) polycot seedlings from an S2 tricot line (CN107316-2Ⓣ11). (**A**–**C**) was obtained from various S0 plants, while (**D**) from the S1 tricot (CN102136-Ⓣ4) seedlings.

We also examined the tricot seedling growth (Figure 1A–C) and only the growth of the first identified tricot seedling was presented in Table 1. Only 15–20% of the tricot seedlings grew normally, 30–40% with intermediate depression, and 20–30% with major depression or death, across three sets of evaluation (Table 1). Thus, only a minority of detected tricot seedlings grew normally to bear seeds, while the majority displayed depression in growth and did not produce seeds. Such variations in seedling growth were largely expected when compared to those reported by Bexon [18] and were compatible with those reports on other species [2]. It is possible that the abnormal growth was the consequence of deleterious alleles acting on the different stages of plant growth and some alleles may even be lethal upon the growth conditions (Table 1). It is also possible that some of the depressed growth or death may reflect the inbreeding depression caused by genes irrelevant to tricot after selfing.

The effort to self the surviving tricot plants resulted in 31 plants bearing S1 seeds, but only 17 S1 lines from 11 accessions had 50 seeds or more (Table 2). Planting seeds of these 17 S1 lines in the greenhouse revealed that the germination rates ranged from 42.5 to 95.0% with an average of 84.9%. The occurrence frequency of tricots per line ranged from 0 to 26.3% and averaged 9.4% of the germinated seeds. There was one S1 line (CN102140-Ⓣ3) producing no tricots with 188 seeds germinated, three lines (CN102136-Ⓣ1; CN102136-Ⓣ4; CN102198-Ⓣ1) having tetracots, and one line (CN102136-Ⓣ4) having 23 S1 plants with chlorosis (Figure 1D).

Continuing to self S1 plants for S2 progeny generated 49 S2 tricot lines and 2 S2 tetracot lines with 40 or more seeds (Table 3). These allowed for the greenhouse evaluation of S2 progeny segregation concerning tricots and tetracots. Planting these S2 lines along with one replicate S2 line revealed that the seed germination rates ranged from 17.7 to 99.0% and averaged 79.8%. The occurrence frequency of tricots per line ranged from 0 to 48.2% and averaged 10.1% of the germinated seeds. Three S2 lines had no tricots or tetracots, 17 (or 34.7%) S2 tricot lines showing 1 to 28 tetracots, and six tricot and tetracot S2 lines having up to 213 S2 plants with chlorosis. Figure 1E illustrates the observed dicot, tricot, and tetracot seedlings in the S2 tricot line (CN107316-2Ⓣ11). Interestingly, two tetracot S2 lines produced only tricot, but no tetracot, plants.

A Chi-square test for the 3:1 genetic segregation of dicot vs. tricot occurrences in S1 and S2 seedlings revealed the presence of a recessive diallelic genetic locus in one (out of 17) S1 line (Table 2) and six (out of 50) S2 lines (Table 3). This finding also suggested the presence of multiple diallelic loci segregating in a large proportion of S1 and S2 seedlings. Interestingly, the S2 tricot line (CN107316-2-Ⓣ11) had a different test outcome from its replicate (CN107316-2-Ⓣ11-r) (Table 3). Overall, these results indicate the observed polycotyledony in yellow mustard was largely controlled by a combination of genes at multiple diallelic loci. Thus, the genetic inheritance of the yellow mustard polycotyledony is complex. Another interesting result was that the frequency of the recessive allele(s) conditioning tricots varied greatly but can be high for some selfed lines, as some S1 lines had up to 26.3% frequency of tricot occurrence (e.g., CN102136-Ⓣ4) and some S2 lines up to 48.2% (e.g., CN102192-1-Ⓣ10).

Our genetic segregation analysis was preliminary and only suggestive in nature. For example, it was reported that *Arabidopsis* tricots can be caused by abnormal chromosome segregation at meiosis [24]. Tricots were also observed in a stochastic manner in *Arabidopsis* (e.g., see [25]). However, the results of the segregation analyses (Table 2 and Table 3) are consistent with the knowledge acquired earlier about the polygenic inheritance of the tricot trait (e.g., see ref [16,17,18,19,20]). Many loci conditioning the development of polycotyledony in *Arabidopsis* and *Antirrhinum* have been identified [20]. One interesting result is the increase of tricot frequency over three generations as the maximum tricot frequencies observed in S0 seedlings was 11.3% (Table 1), in S1 26.3% (Table 2), and in S2 48.2% (Table 3). A specific example of tricot increase was 3% for S0 CN102192 (Table 1) and up to 48.2% in its S2 tricot lines (Table 3). Such increased tricot frequencies were compatible with those reported over the generations of selecting polycotyledons in several species (e.g., see [19,26,27]). Thus, this finding has some implications for the tricot line development for yellow mustard breeding (e.g., [6]) and genetic, genomic, and molecular studies of tricots [10]. Further research is desirable to assess if tricots are associated with seedling establishment, seed yield, oil quality, and other agricultural traits for a possible agricultural benefit [12].

## 3. Methods and Materials

The study materials included 132 yellow mustard accessions with seeds available for distribution that were obtained in April 2013 from the yellow mustard collection maintained at Plant Gene Resources of Canada. The acquired seeds of each accession were randomly separated into three sets: the first set with 54 seeds, the second set with 36 seeds, and the third set with the remaining seeds (ranging from 0 to 108 seeds). The first set was planted in May 2013 in the Saskatoon Research and Development Centre greenhouse. The layout was based on 9 × 4 planting trays, with 4 accessions per tray, and 6 seeds in each of the 36 cells. The soil mix consisted of a regular soilless mix [28] blended with coconut fiber. The greenhouse conditions were 22 °C during the day and 16 °C at night, with a photoperiod of 16 h between 4 am and 8 pm. Germination was recorded on the 8th day. Tricot and tetracot seedlings were identified, counted, and evaluated according to their growth patterns (normal growth, intermediate depression, major depression, and chlorosis). Surviving S0 tricot and tetracot plants were transplanted into 6-inch pots and bagged before flowering to generate selfed S1 progeny. Seeds were collected from each tricot plant and labeled separately for S1 tricot lines. These greenhouse and evaluation procedures were repeated for the second and third sets in October 2013.

To assess the genetic segregation of tricot seedlings, efforts were made from January to August 2014 to evaluate S1 progeny in the greenhouse following the same evaluation procedures as described above. Those surviving S1 tricot and tetracot plants were also bagged to generate selfed S2 progeny. Seeds from each S1 line were collected and labeled separately for S2 tricot lines. Further efforts were made in August 2014 and April 2015 to evaluate the genetic segregation of tricots and tetracots in the S2 progeny. The collected data of tricot and tetracot occurrences in the original S0 generation, S1, and S2 progeny were summarized and analyzed concerning growth patterns and genetic segregation.

A Chi-square test was applied to infer a recessive diallelic genetic locus by assuming the 3:1 genetic segregation of dicot vs. tricot (and tetracot if existed) occurrences in S1 and S2 progeny. A non-significant test (or *p* > 0.05) with one degree of freedom for the critical value of 3.84 means that the tricot (and tetracot) allelic segregation follows a recessive genetic locus.

## 4. Conclusions

The present characterization generated a set of yellow mustard germplasm with frequent occurrences of polycotyledony and novel results on the polygenic inheritances of yellow mustard tricots and tetracots. The germplasm accessions in which we observed the polycotyledons occurring in various frequencies (Table 1, but not those lines derived from selfing) can be acquired for research and breeding via PGRC germplasm requests (https://pgrc-rpc.agr.gc.ca/gringlobal/search; accessed on 8 September 2023). It could be used to facilitate the genetic and/or genomic analysis of polycotyledonous germplasm for a better understanding of genetic and developmental mechanisms conditioning polycotyledony and the development of yellow mustard lines for explorable tricot breeding.

## Figures and Tables

**Table 1:** Observed frequencies of tricot in 46 yellow mustard accessions and the first tricot seedling growth from three sets of greenhouse assessments. CN = Canadian National accession number in the PGRC collection; Origin = country (and region) of origin. Country code follows the ISO code except for UNK (=unknown). Region code in parentheses consists of 1 (North America), 2 (Asia), 3 (East Europe), 4 (West Europe) and 5 (other regions and/or unknown); Type = yellow (Y) and/or brown (B) seed color of an accession known to be either a breeding line/cultivar (C) or a wild collection/landrace (L); GR(54) or GR(36) = germinate rate from 54 or 36 seeds planted; TC = tricot count; 1TG = the first tricot seedling growth; SP = seeds planted; TP = tricot percentage.

				Set 1			Set 2				Set 3			
CN	Description	Origin	Type	GR(54)	TC	1TG	GR(36)	TC	1TG	SP	GR(sp)	TC	1TG	TP
102136	Trico	SWE(4)	Y/C	63.0	3	−2	66.7	2	0	36	61.1	4	−2	11.3
102152	Yade	UNK(5)	Y/C	72.2	1	0	88.9	1	died	45	71.1	4	died	5.8
107311	Galben de Craiova	ROM(3)	Y/C	88.9	2	−1	86.1	1	died	3	66.7			3.7
102135	SRS 527	UNK(5)	Y/L	90.7	4	−2	91.7			35	80.0			3.6
40230	PGR 13245	RUS(3)	Y/L	96.3	1	−1	91.7	1	−1	46	93.5	2	−2	3.1
102192	CR 1816/83	RUS(3)	Y/L	79.6	2	−1	75.0			35	85.7	1	−1	3.0
30473	VNIIMK 162	RUS(3)	Y/L	94.4			86.1	1	died	54	59.3	2	−1	2.6
107309	Mirly	AUT(4)	Y/C	94.4	2	−1	88.9	1	−1	48	83.3			2.4
102170	Serval	NLD(4)	Y/C	64.8			83.3	2	−2	41	41.5			2.4
102201	CR 2044/92	GRC(3)	Y/L	94.4	3	0	94.4			48	91.7			2.3
102193	CR 1835/94	MNG(2)	Y/L	96.3			86.1	3	−1	59	78.0			2.3
102188	CR 354/79	RUS(3)	Y/L	96.3	3	−1	94.4			56	80.4			2.3
102146	Steinacher	DEU(4)	Y/C	64.8			86.1	1	died	36	61.1	1	died	2.3
102168	Santa Fe	UNK(5)	Y/C	79.6			77.8			25	72.0	2	−2	2.2
102137	Trico “elite 70”	SWE(4)	Y/C	64.8			86.1	1	−1	36	77.8	1	−1	2.1
102138	PI 312848	SWE(4)	Y/L	79.6	2	−2	94.4			35	82.9			1.9
102166	Martigena	UNK(5)	Y/C	88.9			97.2	1	died	31	96.8	1	−2	1.8
107316	Perine	FRA(4)	Y/C	100.0	1	−1	72.2			60	58.3	1	−2	1.7
102174	Ascot	UNK(5)	Y/C	61.1	1	−1	63.9			5	40.0			1.7
102199	CR 2031/89	GRC(3)	Y/L	96.3			94.4			44	90.9	2	−2	1.6
107315	Kastor	DEU(4)	Y/C	100.0	1	−1	77.8	1	−2	48	97.9			1.6
102140	Prerovska Bila	HUN(3)	Y/C	61.1	1	−2	93.8			NS	NS			1.5
102200	CR 2032/90	ITA(4)	Y/L	74.1	1	−1	52.8			18	61.1			1.4
102198	CR 2030/79	GRC(3)	Y/L	100.0			83.3	1	−1	60	95.0	1	−1	1.4
102151	Mansholt	NLD(4)	Y/C	53.7	1	−2	52.8			36	63.9			1.4
102171	Condor	UNK(5)	Y/C	70.4	1	0	88.9			7	71.4			1.3
102149	Albatross	DEU(4)	Y/C	66.7			88.9	1	−1	15	46.7			1.3
102167	Salvo	UNK(5)	Y/C	70.4	1	−1	75.0			28	60.7			1.2
107310	Valiant	UNK(5)	Y/C	96.3	1	0	86.1			NS	NS			1.2
102134	Dialba	FRA(4)	Y/C	79.6			86.1			10	90.0	1	1	1.2
102169	Ultra	UNK(5)	Y/C	79.6	1	−2	77.8			40	67.5			1.0
107317	Waldmanns halloren	DEU(4)	Y/C	98.1			69.4			24	91.7	1	0	1.0
107302	Andante	CAN(1)	Y/C	96.3	1	0	91.7			21	95.2			1.0
102187	CR 350/95	DEU(4)	Y/L	98.1	1	−1	86.1			31	87.1			0.9
107314	Maleksberger Gelb	DEU(4)	Y/C	98.1	1	−1	66.7			46	78.3			0.9
107304	TMP 11943	ITA(4)	B/L	98.1	1	−2	91.7			46	60.9			0.9
102191	CR 541/79	RUS(3)	Y/L	90.7			86.1	1	−1	59	57.6			0.9
102208	CR 2086/95	CAN(1)	Y/L	94.4	1	−1	66.7			48	83.3			0.9
102207	CR 2073/95	LTU(3)	Y/L	98.1			91.7	1	−2	32	96.9			0.9
43449	PGR 16791	ESP(4)	Y/L	90.7	1	0	88.9			41	90.2			0.8
102155	WIR 239	RUS(3)	Y/L	90.7			97.2			44	79.5	1	−1	0.8
102156	WIR 1941	RUS(3)	Y/L	79.6			91.7			47	91.5	1	−1	0.8
102196	CR 2028/82	BGR(3)	Y/L	96.3			88.9			62	91.9	1	−1	0.7
33056	Commercial Yellow	CAN(1)	Y/C	92.6	1	0	91.7			72	84.7			0.7
102197	CR 2029/82	RUS(3)	Y/L	94.4			94.4			76	94.7	1	−2	0.6
102194	CR 1836/94	RUS(3)	BY/L	94.4			94.4			99	85.9	1	−1	0.6
*Mean or total*				85.4	40		84.0	20			77.2	29		1.9
*1st tricot seedling growth*														
Normal (0)						7			1				1	
Intermediate depression (−1)						13			7				8	
Major depression (−2)						7			3				7	
Died						0			5				2	

**Table 2:** Observed frequencies of tricot and tetracot seedlings and non-significant Chi-square tests of expected 3:1 segregation ratio for a diallelic locus in S1 seedlings of 17 selfed lines generated from 11 yellow mustard accessions.

Self-Line Label	S1 Seeds Planted	S1 Seeds Germinated	Germination Rate (%)	Tricot Count	Tricot (%)	Tetracot Count	S1 Plants with Chlorosis	3:1 Segregation Ratio Test
CN102136-Ⓣ1	153	65	42.5	5	7.7	1	0	Not 3:1
CN102136-Ⓣ2	129	115	89.1	23	20.0	0	0	Not 3:1
CN102136-Ⓣ4	200	156	78.0	41	26.3	4	23	3:1
CN102137-Ⓣ1	143	117	81.8	2	1.7	0	0	Not 3:1
CN102140-Ⓣ3	200	188	94.0	0	0.0	0	0	Not 3:1
CN102152-Ⓣ2	145	132	91.0	3	2.3	0	0	Not 3:1
CN102152-Ⓣ3	80	76	95.0	14	18.4	0	0	Not 3:1
CN102166-Ⓣ1	200	173	86.5	22	12.7	0	0	Not 3:1
CN102170-Ⓣ2	64	59	92.2	5	8.5	0	0	Not 3:1
CN102170-Ⓣ1	200	183	91.5	5	2.7	0	0	Not 3:1
CN102191-Ⓣ1	151	129	85.4	15	11.6	0	0	Not 3:1
CN102193-Ⓣ1	200	167	83.5	26	15.6	0	0	Not 3:1
CN102193-Ⓣ2	185	174	94.1	9	5.2	0	0	Not 3:1
CN102194-Ⓣ1	200	183	91.5	11	6.0	0	0	Not 3:1
CN102196-Ⓣ1	88	69	78.4	2	2.9	0	0	Not 3:1
CN102198-Ⓣ1	200	148	74.0	22	14.9	10	0	Not 3:1
CN102198-Ⓣ2	200	190	95.0	6	3.2	0	0	Not 3:1

**Table 3:** Observed frequencies of tricot and tetracot seedlings and non-significant Chi-square tests of expected 3:1 segregation ratio for a diallelic locus in S2 seedlings of 50 selfed tricot (including one replicate) and 2 tetracot lines generated from 13 yellow mustard accessions.

Self-Line Label	S2 Seeds Planted	S2 Seeds Germinated	Germination Rate (%)	Tricot Count	Tricot (%)	Tetracot Count	S2 Plants with Chlorosis	3:1 Segregation Ratio Tests
*Tricot*								
CN102136-1-Ⓣ1	120	89	74.2	7	7.9	3	0	Not 3:1
CN102136-4-Ⓣ1	200	169	84.5	7	4.1	0	34	Not 3:1
CN102136-4-Ⓣ2	88	77	87.5	10	13.0	0	14	Not 3:1
CN102136-4-Ⓣ5	200	182	91.0	14	7.7	1	34	Not 3:1
CN102136-5-Ⓣ1	200	187	93.5	25	13.4	2	0	Not 3:1
CN102136-5-Ⓣ5	400	350	87.5	47	13.4	2	0	Not 3:1
CN102136-5-Ⓣ6	296	276	93.2	55	19.9	0	0	Not 3:1
CN102138-3-Ⓣ3	200	180	90.0	2	1.1	0	0	Not 3:1
CN102138-3-Ⓣ5	400	319	79.8	6	1.9	0	0	Not 3:1
CN102138-3-Ⓣ7	400	321	80.3	16	5.0	0	0	Not 3:1
CN102138-3-Ⓣ8	400	373	93.3	22	5.9	0	0	Not 3:1
CN102138-3-Ⓣ9	200	186	93.0	9	4.8	0	0	Not 3:1
CN102149-1-Ⓣ1	144	108	75.0	18	16.7	4	0	Not 3:1
CN102152-1-Ⓣ4	400	387	96.8	18	4.7	0	0	Not 3:1
CN102152-1-Ⓣ6	400	346	86.5	6	1.7	0	0	Not 3:1
CN102152-1-Ⓣ7	200	56	28.0	3	5.4	0	0	Not 3:1
CN102152-1-Ⓣ8	400	354	88.5	23	6.5	0	213	Not 3:1
CN102167-1-Ⓣ2	280	270	96.4	7	2.6	1	0	Not 3:1
CN102167-1-Ⓣ3	400	385	96.3	2	0.5	0	0	Not 3:1
CN102167-1-Ⓣ4	184	112	60.9	3	2.7	0	0	Not 3:1
CN102167-1-Ⓣ5	400	183	45.8	4	2.2	0	0	Not 3:1
CN102167-1-Ⓣ6	400	344	86.0	5	1.5	1	0	Not 3:1
CN102167-1-Ⓣ7	392	388	99.0	7	1.8	0	0	Not 3:1
CN102167-1-Ⓣ8	400	381	95.3	14	3.7	0	0	Not 3:1
CN102169-1-Ⓣ1	200	194	97.0	0	0.0	0	0	Not 3:1
CN102171-2-Ⓣ1	200	185	92.5	5	2.7	0	0	Not 3:1
CN102187-2-Ⓣ1	195	158	81.0	2	1.3	0	0	Not 3:1
CN102187-3-Ⓣ1	200	194	97.0	0	0.0	0	0	Not 3:1
CN102187-4-Ⓣ1	280	265	94.6	1	0.4	0	0	Not 3:1
CN102187-4-Ⓣ2	384	348	90.6	0	0.0	0	0	Not 3:1
CN102187-4-Ⓣ3	200	173	86.5	3	1.7	0	0	Not 3:1
CN102192-1-Ⓣ3	200	134	67.0	40	29.9	10	0	3:1
CN102192-1-Ⓣ5	144	55	38.2	20	36.4	3	0	3:1
CN102192-1-Ⓣ8	93	48	51.6	4	8.3	1	0	Not 3:1
CN102192-1-Ⓣ10	96	56	58.3	27	48.2	5	0	3:1
CN102192-1-Ⓣ11	188	65	34.6	22	33.8	5	0	3:1
CN102192-1-Ⓣ14	288	51	17.7	8	15.7	0	0	Not 3:1
CN102192-1-Ⓣ15	192	150	78.1	29	19.3	2	0	Not 3:1
CN102192-1-Ⓣ17	216	158	73.1	20	12.7	0	0	Not 3:1
CN102192-1-Ⓣ20	124	60	48.4	21	35.0	14	0	3:1
CN102201-1-Ⓣ1	46	35	76.1	5	14.3	0	0	Not 3:1
CN102206-1-Ⓣ1	68	58	85.3	1	1.7	0	0	Not 3:1
CN102208-1-Ⓣ1	152	142	93.4	3	2.1	0	0	Not 3:1
CN107316-2-Ⓣ1	144	124	86.1	10	8.1	0	0	Not 3:1
CN107316-2-Ⓣ2	196	187	95.4	24	12.8	2	0	Not 3:1
CN107316-2-Ⓣ4	70	63	90.0	1	1.6	0	0	Not 3:1
CN107316-2-Ⓣ5	119	107	89.9	3	2.8	0	0	Not 3:1
CN107316-2-Ⓣ6	177	150	84.7	3	2.0	0	0	Not 3:1
CN107316-2-Ⓣ11	200	179	89.5	71	39.7	28	0	3:1
CN107316-2-Ⓣ11-r	392	334	85.2	81	24.3	28	82	Not 3:1
*Tetracot*								
CN102136-4-④1	200	134	67.0	9	6.7	0	32	Not 3:1
CN102152-1-④12	43	42	97.7	7	16.7	0	0	Not 3:1

## Data Availability

The data presented in this study are available in Supplementary Materials.

## Supplementary Materials

The following supporting information can be accessed from Figshare DOI (https://doi.org/10.6084/m9.figshare.24114597). Table S1: The list of 132 yellow mustard accessions and their observed frequencies of tricots.
